# Long-term cardiac assessment in a sample of adolescent-onset anorexia nervosa

**DOI:** 10.1186/s40337-022-00533-w

**Published:** 2022-01-31

**Authors:** I. Flamarique, B. Vidal, M. T. Plana, S. Andrés-Perpiñá, M. Gárriz, P. Sánchez, C. Pajuelo, L. Mont, J. Castro-Fornieles

**Affiliations:** 1 Department of Child and Adolescent Psychiatry and Psychology, 2017SGR881, Clinic Institute of Neurosciences, Hospital Clínic Universitari de Barcelona, IDIBAPS, CIBERSAM, C/Villarroel, 170, 08036 Barcelona, Spain; 2 Institut Clínic Cardiovascular, Hospital Clínic de Barcelona, Institut d’Investigacions Biomèdiques August Pi i Sunyer, Barcelona, Spain; 3 Instituto de Salud Carlos III (CB16/11/00354), CIBERCV, Madrid, Spain; 4 Institute of Neuropsychiatry and Addictions, Parc de Salut Mar de Barcelona, Llull, 410, 08019 Barcelona, Spain; 5 Department of Cardiology, Hospital Plató, Barcelona, Spain; 6 Department of Medicine, University of Barcelona, Barcelona, Spain

**Keywords:** Anorexia nervosa, Cardiac assessment, Adolescent, Long-term follow-up, Longitudinal study

## Abstract

**Background:**

High mortality rates have been reported in patients with anorexia nervosa, mainly due to cardiovascular alterations. The purpose of the present study was to assess cardiac structural and functional abnormalities some 20 years after initial treatment in a sample of adolescent-onset anorexia nervosa (A-AN) and to compare them with matched healthy controls (HC).

**Methods:**

A sample of 29 women diagnosed and treated for AN during adolescence (A-AN) were assessed more than 20 years later. A complete cardiac evaluation was carried out including an electrocardiogram (ECG) and a standard 2D echocardiography. Thirty matched HC were also assessed.

**Results:**

In the A-AN group, four subjects had a body mass index lower than 18.5 and met full DSM 5 criteria for AN at follow-up (Low-Weight group). They were compared with the rest of the sample (n = 25) who had normalized their weight (Normal-Weight group), though some still showed some eating disorder symptoms. Both groups were compared with the HC group. Subjects in the Low-Weight group presented statistically significant decreases in the left ventricular end-diastolic and left atrium dimensions and left ventricular mass in comparison with the Normal-Weight group and the HC. No other differences in cardiac parameters were found between groups.

**Conclusions:**

Echocardiographic and ECG parameters of adults who had presented A-AN twenty years earlier and currently maintained normal weight were similar to those of HC who had never been treated or diagnosed with AN. Adult subjects with A-AN who still had low weight in the long term present certain cardiac abnormalities similar to those seen in short-lasting disease. More studies are needed to confirm these results in a larger sample.

## Plain English summary

Anorexia nervosa is associated with multiple medical complications and high mortality, mainly due to cardiovascular complications. The main objective of the project was to study long-term cardiac abnormalities in a group of patients diagnosed with anorexia nervosa during adolescence. A sample of 29 patients, treated during adolescence for anorexia nervosa, were evaluated 20 years later. We did an echocardiogram and an electrocardiogram to all of them, and compared them with 30 healthy controls. Of the 29 patients with anorexia nervosa, 4 had low weight and 25 had normal weight. Patients who had normalized their weight did not present cardiac alterations and did not differ from the healthy controls. The 4 underweight patients did present cardiac abnormalities similar to those observed in short-term studies, such as decreased dimensions and mass of the left ventricle and the left atrium.

## Introduction

High mortality rates have been reported in patients with anorexia nervosa (AN), largely due to medical complications associated with poor nutritional status [1]. Among all medical abnormalities, cardiovascular alterations have been related to at least one-third of all mortality in patients with AN, mainly due to sudden death [2, 3]. The most common cardiovascular complications are sinus bradycardia and hypotension [4–7].

Among cardiac structural alterations, previous studies in AN patients have shown abnormalities including mitral valve prolapse, mild pericardial effusion, reduced left ventricular (LV) mass, cardiac output, LV end-diastolic (LVEDD) and end-systolic (LVESD) dimensions and left posterior wall thickness [4–6, 8–10]. Despite reduced LV mass and dimensions, in some studies, ventricular systolic function has been found to be similar to that in female controls [8–11].

With regard to functional changes, prolonged QT and greater QT dispersion have been found in some studies with AN patients [4–6]. These findings are relevant, given that QT dispersion and prolongation have been reported to be markers of severe ventricular arrhythmias and sudden death [12]. However, other studies do not support these findings [7, 13, 14]. A recent study on a large population of patients with eating disorders found that population-mean QT was normal. Marked QTc prolongation occurred solely in the presence of extrinsic factors such as hypokalemia [15]. Galetta et al. [4] found greater QT dispersion in females with AN than in constitutionally thin women or normal weight women; they also confirmed the findings of previous studies that had reported a reduction in left ventricular (LV) mass in AN with echocardiography [5]. These authors suggested that the relationship between QT dispersion and LV mass index in the starvation phase of AN might be the factor responsible for the increased QT dispersion [4].

Some authors have studied the reversibility of cardiovascular abnormalities in adolescents with AN after refeeding in the short term. In 31 adolescents with AN at admission and after refeeding (3–18 months), Mont and Castro [16] observed a normalization of cardiac structural and functional abnormalities after weight recovery. Similar results were observed in a sample of 40 adolescents with AN by Olivares et al. [6], who concluded that weight gain after 6–18 months was associated with cardiac improvement. A retrospective study by Kastner et al. [10] that included 143 adolescents with AN did not find complete reversibility of echocardiography parameters; the authors attributed the differences in their findings with respect to the previous studies to the shorter duration of refeeding (1–6 months approximately) and the lower weight gain. Therefore, it is important to clarify if cardiovascular abnormalities in AN are reversible in the long term, mainly because they are associated with a significant percentage of all mortality in patients with AN [2, 3]; but also because not all medical complications have been proven to be fully reversible, such as is the case with low bone mineral density [17].To our knowledge, no studies to date have assessed cardiac functional and structural abnormalities of adolescent-onset AN (A-AN) in the long term. The aim of the present study is to study cardiac structural and functional abnormalities some 20 years after initial treatment in a sample of A-AN.

## Methods

### Sample

A sample of women who were diagnosed and treated for anorexia nervosa during adolescence (A-AN) at the Department of Child and Adolescent Psychiatry and Psychology of the Hospital Clinic of Barcelona between 1987 and 1993 (n = 62) were contacted. Thirty-eight agreed to participate in the follow-up study, which consisted of a complete comprehensive assessment including clinical, global functioning, personality, neuroimaging, biochemical, hormonal and cardiac data. Some results of the follow-up study have already been published [18–20]. The present study focused on the cardiac assessment. The mean number of years since first assessment at the department was 22 (range 17–29 years) [18]. Of the 38 patients who agreed to take part in the follow-up, 29 agreed to participate in the present study.

A comparison group of 30 women of similar age and socio-cultural background was assessed. Exclusion criteria for this group were a history of eating disturbances, severe somatic illness including cardiac severe diseases, and the presence of any medical condition that might seriously affect their body image. Informed consent was obtained from all participants in the study, and data collection and procedures were approved by the Ethics Committee of our institution.

### Clinical data during adolescence in former A-AN patients

The clinical records of women with A-AN who agreed to participate in the study were closely examined, and clinical characteristics and body mass index (BMI) at that time were recorded.

### Clinical and cardiac assessment at twenty-year follow-up

#### Clinical data

All participants were interviewed using the Structured Clinical Interview for Axis I Disorders—Spanish version (SCID-I) [21]. The SCID-I was used to determine the possible diagnoses that subjects present at the current time. Weight and height were measured and Body Mass Index (BMI) was calculated for all participants. Participants were also asked about medical conditions.

#### Cardiac evaluation

A cardiac evaluation with a standard 12-lead electrocardiogram (ECG) was carried out. All patients included were also scanned with a standard 2D echocardiography following the American Society of Echocardiography guidelines [22]. Diameters and ejection fraction were measured and LV mass was calculated according to the Penn Convention [23]. All measurements were indexed according to body surface area and weight. Body surface area (BSA) was calculated using the DuBois formula BSA (m2) = 0.20247 (height)0.725 (weight)0.425, with height measured in metres and weight in kilograms [24].

Left ventricular end-diastolic diameter (LVEDD), left ventricular end-systolic diameter (LVESD) and left atrium diameters were measured with M mode in the long axis parasternal view, left ventricular ejection fraction (LVEF) and volumes were quantified with the Simpson method in the 4- and 2-chamber apical view, and diastolic function was assessed studying the trans mitral flow with pulsed wave Doppler in accordance with the recommendations of the American Society of Echocardiography [25]. Fractional shortening was calculated to measure LV contractility using the formula 100 * (LVEDD-LVESD)/LVEDD. Standardization for BSA was performed by dividing the LV mass by the BSA to give the LV mass index. Normal values of LV mass index used were obtained from the study by Cain et al. [26]. Finally, global longitudinal strain (GLS) of the left ventricle was assessed using speckle tracking echocardiography software on 2D gray scale images obtained from the apical 4 and 2-chambers views.

### Statistical analysis

Descriptive summary statistics [means, median, 25th–75th percentiles, standard deviations (SD), frequencies, percentages] were used to describe the sociodemographic and clinical characteristics of the sample. The sample was divided in three groups for the purposes of the present study. The Kruskal Wallis tests and Dunn non-parametric post-hoc method were used to compare clinical data, echocardiographic and ECG data between the three groups. All statistical analyses were performed using SPSS v23.0 for Windows.

## Results

### Clinical and sociodemographic data during adolescence of A-AN patients

Based on DSM-5 criteria, the diagnosis at initial assessment during adolescence in the 29 patients were: atypical AN in two patients (3.4%), mild AN (BMI ≥ 17 kg/m^2^) in six patients (10.2%) moderate AN (BMI 16–16.99 kg/m^2^) also in six patients (10.2%), severe AN (BMI 15–15.99 kg/m^2^) in five (8.5%) and extreme AN (BMI ≤ 15 kg/m^2^) in ten (16.9%).

Mean age at onset was 13.58 (Standard deviation SD 1.4) years and patients had their first contact with our department at age 14.5 (SD 1.6) years. Mean BMI at their initial assessment was 15.9 kg/m^2^ (range 10–24; SD 2.6), and the mean percentile of BMI was 10.8 (range 10–80; SD 17.09), with a mean weight loss of 12.82 kg (range 1–30; SD 6.06).

### Comparison between former A-AN patients and Healthy Controls regarding clinical characteristics at follow-up

At the follow-up evaluation, most of the former A-AN group did not meet DSM-5 criteria for any eating disorder (n = 18; 62%). Four subjects (13.8%) met full DSM-5 criteria for AN, three (10.3%) met full DSM-5 criteria for atypical AN, and two (6.9%) met DSM-5 criteria for atypical BN. Two patients (6.9%) showed partial remission of AN (physical recovery, but persistent fear of gaining weight or body image disturbance). The former A-AN subjects were divided into two groups according to their weight status: the Low-Weight group, which included the four patients (6.8%) who fulfilled DSM-5 criteria for AN and had a BMI < 18.5 [27], and the Normal-Weight group (n = 25; 86.2%), with a current BMI above 18.5 (including patients who still met criteria for an eating disorder (ED) and patients who showed a complete remission of the ED. In the present study, the Low-Weight group (n = 4); Normal-Weight group (n = 25) and Healthy controls (HC) (n = 30) were compared.

Data regarding the comparison of clinical characteristics groups are shown in Table 1. Mean BMI was 14.8 kg/m^2^ (SD = 1.95) in the Low-Weight group, 21.5 (SD = 2.19) in the Normal-Weight group and 22.19 kg/m^2^ (SD = 2.19) for the HC. There were no differences in height or age between groups. Nine subjects were taking medication. In the Low-Weight group three (75%) were taking antidepressants, two (50%) were also taking antipsychotics (olanzapine) and one (25%) benzodiazepines. In the Normal-Weight group, five subjects (20%) were taking antidepressants, two (8%) were taking benzodiazepines, one (4%) was also taking an antipsychotic (olanzapine), and one (4%) was taking lithium. Only one subject (3.3%) in the control group was taking medication (lorazepam).One participant had been diagnosed with epilepsy and another one with hypothyroidism. None of them had hypertension or obesity.

**Table 1:** Comparison of clinical data between groups

	Low-weight AN group Median (25th–75th percentiles) (n = 4)	Normal-weight AN group Median (25th–75th percentiles) (n = 25)	Healthy controls Median (25th–75th percentiles) (n = 30)	H^a^	*p*	Dunn post-hoc test^b^
BMI (kg/m^2^)	14.95 (12.9–16.6)	21.35 (20.17–22.89)	22.01 (20.36–23.72)	11.865	0.003*	LW < NW*@LW < HC*
Height (m)	1.60 (1.56–1.67)	1.62 (1.58–1.68)	1.65 (1.58–1.69)	1.484	0.476	
Age (years)	35.5 (30.25–42.25)	36 (34–37)	36 (34.75–39.25)	0.866	0.648	

Bold: groups that show statistically significant differences

Bold and italics: statistically significant

*LW* low-weight group, *NW* normal-weight group, *HC* healthy controls

*p < 0.05

^a^Kruskal Wallis test

^b^Dunn–Bonferroni post hoc test; Statistical significant *p* < 0.05

### Comparison of echocardiographic data between groups

Echocardiographic data of the groups are compared in Table 2. Patients in the Low-Weight group presented statistically significant decreases in the LVEDD, LV mass and left atrium dimensions in comparison with the Normal-weight group and the Healthy Controls. The Low-Weight group showed a reduced LV mass indexed by BSA compared with the other groups, the difference almost reaching statistical significance. The percentage of patients with low LV mass indexed by BSA was higher in the Low-weight group (50%) than in the Normal-Weight group (4.2%) and the HC (17.2%) (χ^2^ = 6.47, *p* = 0.039). No differences were found between the Low-weight group and the HC and Normal-weight group for other cardiac parameters including left ventricular systolic function neither measured by the Simpson’s method nor by GLS. One patient in the Low-Weight group presented mild tricuspid regurgitation and another had a mild dilated right ventricle.

**Table 2:** Comparison of echocardiographic data between groups

	Low-weight AN group Median (25th–75th percentiles) (n = 4)	Normal-weight AN group Median (25th–75th percentiles) (n = 25)	Healthy controls Median (25th–75th percentiles) (n = 30)	H^a^	*p*	Dunn post-hoc test^b^
LVEDD (mm)	40.75 (36.87–42)	47.37 (43.69–48.3)	46.47 (43.22–48)	9.741	***0.008***	^**a**^LW < NW* LW < HC*
LVESD (mm)	23.8 (21.4–25.75)	26.9 (25–30)	28.55 (24.92–31.56)	5.415	0.067	
IVSD (mm)	6.1 (4.77–6.75)	7 (6.25–8.08)	6.9 (6.17–7.52)	3.640	0.162	
LVPWDd (mm)	5.55 (4.5–6.07)	5.8 (5.15–6.64)	6 (5.1–6.92)	1.649	0.438	
Left atrium (mm)	27.5 (24.75–28.75)	30 (29–33)	32 (30–34)	7.792	***0.020***	^**a**^LW < HC*
LV mass (g)	66.4 (45.85–96.17)	113.15 (105.3–134.7)	120.4 (97.2–140.3)	8.290	***0.016***	^**a**^LW < NW* LW < HC*
LV mass Index (g/m^2^)	50.47 (36.38–64.95)	72.97 (65.22–80.35)	73.11 (61.69–83.28)	5.704	0.058	
Fractional shortening (%)	41.07 (38.69–42.50)	41.86 (36.86–44.68)	38.31 (34.1–42.51)	2.701	0.259	
A wave (cm/secs)	42 (34.17–53.57)	42 (38–54)	50.35 (41.5–61)	3.330	0.189	
E wave (cm/secs)	71 (53.1–85.07)	75 (62.97–81.25)	77 (68–92.6)	2.179	0.336	
E/A	1.61 (1.3–2.05)	1.55 (1.27–2.02)	1.5 (1.26–2.09)	0.501	0.778	
LVEF Simpson (%)	62 (58.25–68)	60 (57.5–67.75)	63 (60–65)	0.560	0.756	
GLS (%)	− 24 (− 24)	− 22 (− 20, − 23)	− 22 (− 20, − 24)	3.033	0.219	

Bold: groups that show statistically significant differences

Bold and italics: statistically significant

*LV* left ventricular, *LVEDD* LV end-diastolic diameter, *LVESD* LV end-systolic diameter, *IVSD* interventricular septal wall dimension, *LVPWDd* LV posterior wall dimension, *LVEF* left ventricular ejection fraction, *GLS* global longitudinal strain, *LW* low-weight group, *NW* normal-weight group, *HC* healthy controls

*p < 0.05

^a^Kruskal Wallis test

^b^Dunn–Bonferroni post hoc test; Statistical significance *p* < 0.05

As for the comparison between groups with normal weight (the Normal-Weight group and the HC), no differences were observed in any echocardiographic measures or in systolic or diastolic parameters.

### Comparison of electrocardiographic data between groups

Data regarding electrocardiographic parameters are shown in Table 3. Only one patient (25%) in the Low-Weight group presented bradycardia. None of the patients in the Low-Weight group showed prolonged QT or QTc. No differences were observed between groups regarding heart rate, QRS width, PR interval, QT or QTc interval and QT dispersion.

**Table 3:** Comparison of electrocardiographic data between groups

	Low-weight AN group Median (25th-75th percentiles) (n = 4)	Normal-weight AN group Median (25th-75th percentiles) (n = 25)	Healthy Controls Median (25th-75th percentiles) (n = 30)	H^a^	p	Dunn Post-hoc test^b^
Heart rate (beats/min)	65 (56.75–85.25)	65 (54–75)	68.5 (59.75–74.50)	0.717	0.699	
QRS axis degree	60 (-7.50–82.50)	60 (30–60)	60 (60–60)	0.084	0.959	
Width of the QRS (msec)	80 (80–87.50)	80 (80–80)	80 (80–80)	3.534	0.171	
PR interval (msec)	140 (110–140)	120 (120–160)	140 (120–160)	0.234	0.890	
QT interval (msec)	380 (345–430)	400 (380–400)	390 (365–400)	0.312	0.856	
Corrected QT (msec)	416.50 (384.50–420)	412 (393–426.50)	410 (394–425)	0.075	0.963	
QT dispersion	25 (12.5–37.5)	20 (12.5–37.5)	20 (12.5–37.5)	0.176	0.916	

*LW* low-weight group, *NW* normal-weight AN group, *HC* healthy controls

^a^Kruskal Wallis test

^b^Dunn–Bonferroni post hoc test; statistical significance *p* < 0.05

## Discussion

The main finding of the present study is the absence of any differences in echocardiographic or ECG parameters between HC and patients with A-AN who, 20 years after their initial treatment, had recovered normal weight. However, patients with A-AN who had low weight at follow up showed cardiac structural abnormalities such as decreases in the LVEDD, LV mass and left atrium dimensions compared to the HC and the Normal-weight group. Moreover, the percentage of patients with low LV mass indexed by BSA in the Low-Weight group was higher than in the Healthy and Normal-Weight group.

In the present study, significant decreases in the LVEDD, LV mass and left atrium was observed in the Low-Weight group compared with the Normal-Weight group and the HC, but no differences were observed for left ventricular systolic function. These results are in line with previous studies which have found structural abnormalities in AN patients but no differences in systolic function compared with controls [4–6, 8–11, 28]. Studying cardiac dimensions and ventricular function in 95 adolescents with AN in comparison with 58 controls, [8] found significant reductions in LV dimensions, LV wall thickness, LV mass and Left atrium dimension in patients with AN [8]. Despite the decreases in LV mass and LV dimensions, resting systolic function in AN patients was unchanged. The differences for the LV mass indexed by BSA between groups did not reach statistical significance; this may have been due to the small sample size in the Low-Weight group, since previous studies with larger samples have reported significant differences [5, 6, 8, 9, 29].

One patient in the Low-Weight group presented mild tricuspid regurgitation. This condition has been described in patients with congestive heart failure and severe malnutrition [30]. Another patient with AN had a dilated right ventricle; however, it has been suggested that normal right ventricular wall thickness and chamber size may give the impression of a dilated right ventricle when compared to the small left ventricle in patients with malnutrition [31].

We found no differences in any cardiac parameter between HC and Normal-Weight groups, all of which were in the normal range. Taken together, these results indicate that although cardiac structural abnormalities have been observed in low-weight patients, they are not found when patients recover normal weight.This could suggest that cardiac abnormalities found in low weight patients could be reversible, in contrast with low bone mineral density that can be a chronic medical problem in this population [17]. Most studies that have assessed the reversibility of cardiac abnormalities in the short term after refeeding have found similar results [5, 6], though not all [10]. Mont and Castro [16] compared cardiac parameters of adolescents with AN before and after refeeding and weight normalization (3–18 months later). After refeeding, these authors observed significant increases in the LVEDD, LVESD, left atrium dimension and the LV mass, all of which returned to normal values in the majority of patients. LVEF was normal before and after refeeding for all subjects. The study by Olivares et al. [6] compared cardiac parameters of 40 young women with AN, at baseline and after renutrition (6–18 months), and matched them with 40 healthy controls. The authors observed a significant reduction in cardiac cavities and in myocardial mass in patients with AN compared with controls; after treatment and weight recovery, myocardial size and cardiac cavities returned to normal after adjustment for body mass [6]. Lastly, Kastner et al. [10] studied 173 adolescents with AN admitted to hospital before and after renutrition and compared them with 40 healthy controls [10], finding that anorexic patients had reduced LVEDD and LVESD in comparison to controls at baseline. They did not observe a relevant increase in LVEDD and LVESD, but the duration of treatment was shorter (1–6 months) and weight gain was lower (mean BMI at recovery 17.12 ± 1.27) than in the other studies that have assessed the reversibility of cardiac abnormalities [5, 6, 10].

Regarding functional changes, we did not find differences between groups in any of the ECG parameters. One patient in the Low-Weight group presented sinus bradycardia, which has been reported as the most frequent arrhythmia in patients with AN [4, 5, 14, 28, 32]. None of the patients in the Low-weight group presented prolonged QTc or QT dispersion, findings recorded by some authors in patients with AN [4–6], though not by others [7, 13, 14]. Potential causes of these discrepancies include electrolytic disturbances, the use of drugs known to prolong QT interval, ionic channel genetics, idiopathic long QT syndrome, metabolic factors, hormones or decrease in LV mass index.

The main limitation of the study is the small sample size, especially in the Low-Weight group; however, since most adolescent-onset AN who receive treatment recover from the illness, this is inevitable [18]. A relevant limitation is the lack of baseline echo and EKG data, so no conclusions regarding recovery can be made. A further limitation is that not all the patients contacted agreed to participate in the study. It is possible that the final sample was composed of patients with a better prognosis or higher motivation to collaborate with studies of this kind. Another limitation is that participants were not asked about smoking habits.

The main strength of the study is that, to our knowledge, it is the first to compare cardiac parameters in the long term between A-AN after 20 years of initial treatment and healthy controls. Another strength is its comparison of the A-AN sample with a matched sample of HC.

## Conclusions

In summary, our study shows that echocardiographic or ECG parameters of adolescents who presented with adolescent-onset AN but have normal weight 20 years after initial treatment are similar to those of healthy controls who have never been treated for AN or diagnosed with this condition. However, adult subjects with A-AN who still have low weight in the long term present some cardiac abnormalities that are similar to those seen in short-lasting disease. More studies are needed to confirm these results in a larger sample.

## Data Availability

The datasets generated and/or analysed during the current study are not publicly available due to ethical restrictions but are available from the corresponding author on reasonable request.

## Abbreviations

AN: Anorexia nervosa
A-AN: Adolescent-onset anorexia nervosa
BMI: Body mass index
BSA: Body surface area
ECG: Electrocardiogram
GLS: Global longitudinal strain
HC: Healthy controls
LV: Left ventricular
LVEDD: Left ventricular end-diastolic dimension
LVEF: Left ventricular ejection fraction
LVESD: Left ventricular end-systolic dimension
SCID-I: Structured Clinical Interview for Axis I Disorders
SD: Standard deviation
